# Pan-American Lancehead Pit-Vipers: Coagulotoxic Venom Effects and Antivenom Neutralisation of *Bothrops asper* and *B. atrox* Geographical Variants

**DOI:** 10.3390/toxins13020078

**Published:** 2021-01-22

**Authors:** Lachlan A. Bourke, Christina N. Zdenek, Edgar Neri-Castro, Melisa Bénard-Valle, Alejandro Alagón, José María Gutiérrez, Eladio F. Sanchez, Matt Aldridge, Bryan G. Fry

**Affiliations:** 1 Toxin Evolution Lab, School of Biological Sciences, University of Queensland, St Lucia, QLD 4072, Australia; l.bourke@uq.net.au (L.A.B.); c.zdenek@uq.net.au (C.N.Z.); 2 Departamento de Medicina Molecular y Bioprocesos, Instituto de Biotecnologa, Universidad Nacional Autónoma de México, Av. Universidad 2001, Cuernavaca, Morelos 62210, Mexico; nericastroedgare@gmail.com (E.N.-C.); melitza61@gmail.com (M.B.-V.); alagon@ibt.unam.mx (A.A.); 3 Instituto Clodomiro Picado, Facultad de Microbiología, Universidad de Costa Rica, San José 11501, Costa Rica; jose.gutierrez@ucr.ac.cr; 4 Laboratory of Biochemistry of Proteins from Animal Venoms, Research and Development Center, Ezequiel Dias Foundation, Belo Horizonte, MG 30510-010, Brazil; eladiooswaldo@gmail.com; 5 MicroPharm Limited, Station Road Ind. Est., Station Road, Newcastle Emlyn, Carmarthenshire SA38 9BY, UK; matt.aldridge@micropharm.co.uk

**Keywords:** *Bothrops*, coagulotoxicity, antivenom neutralisation, antivenom, venom variation

## Abstract

The toxin composition of snake venoms and, thus, their functional activity, can vary between and within species. Intraspecific venom variation across a species’ geographic range is a major concern for antivenom treatment of envenomations, particularly for countries like French Guiana that lack a locally produced antivenom. *Bothrops asper* and *Bothrops atrox* are the most medically significant species of snakes in Latin America, both producing a variety of clinical manifestations, including systemic bleeding. These pathophysiological actions are due to the activation by the venom of the blood clotting factors Factor X and prothrombin, thereby causing severe consumptive coagulopathy. Both species are extremely wide-ranging, and previous studies have shown their venoms to exhibit regional venom variation. In this study, we investigate the differential coagulotoxic effects on human plasma of six venoms (four *B. asper* and two *B. atrox* samples) from different geographic locations, spanning from Mexico to Peru. We assessed how the venom variation of these venom samples affects neutralisation by five regionally available antivenoms: Antivipmyn, Antivipmyn-Tri, PoliVal-ICP, Bothrofav, and Soro Antibotrópico (SAB). The results revealed both inter- and intraspecific variations in the clotting activity of the venoms. These variations in turn resulted in significant variation in antivenom efficacy against the coagulotoxic effects of these venoms. Due to variations in the venoms used in the antivenom production process, antivenoms differed in their species-specific or geographical neutralisation capacity. Some antivenoms (PoliVal-ICP, Bothrofav, and SAB) showed species-specific patterns of neutralisation, while another antivenom (Antivipmyn) showed geographic-specific patterns of neutralisation. This study adds to current knowledge of *Bothrops* venoms and also illustrates the importance of considering evolutionary biology when developing antivenoms. Therefore, these results have tangible, real-world implications by aiding evidence-based design of antivenoms for treatment of the envenomed patient. We stress that these *in vitro* studies must be backed by future *in vivo* studies and clinical trials before therapeutic guidelines are issued regarding specific antivenom use in a clinical setting.

## 1. Introduction

Venomous snakebites across the Americas result in an estimated 652 to 3466 deaths per year [1], with the majority of these snakebites by pit-vipers [2,3,4,5]. *Bothrops* is a genus of pit-viper—commonly known as lanceheads—that is distributed across South and Central America, as well as Mexico. Epidemiological data suggest that across South America, 70–96.6% of envenomings are inflicted by the *Bothrops* species [3,4,5,6]. Most of these bites are from *B. atrox* in Amazon regions [6,7] or *B. asper* in northern South America [8]. Similar values are also reported across Central America, with 50–80% of all snakebites attributed to *B. asper* [8,9]. Epidemiological data on Mexican *B. asper* bites are scarce, although the incidence is approximately 6 bite cases per 100,000 people per year [8]. In a more recent study, however, Chippaux [10] reported 3.3 snakebite cases per 100,000 people per year in Mexico, although the species responsible for these bites is not made clear. Thus, collectively across the Americas, *B. asper* and *B. atrox* may be considered the two species responsible for most medically significant snakebite envenomations.

Coagulopathy resulting from over-activation of the blood clotting cascade is one of the most common clinical manifestations induced by *Bothrops* venoms [7,8,11]. Bites from such coagulotoxic snakes are particularly difficult to treat, as they cause disruption of an integral bodily function: the body’s blood clotting mechanism (the haemostatic system). The formation of a blood clot ultimately results in the production of a fibrin mesh network that traps red blood cells to arrest bleeding [12]. Fibrin is the end-product of a process known as the coagulation cascade. In this cascading series of biochemical events in the blood, a clot is formed through sequential activation of blood coagulation factors (zymogens). In the final stages of this domino-type effect, prothrombin is converted into its active form (thrombin), which then cleaves fibrinogen into fibrin strands that cross-link to form a blood clot [12].

Numerous snake venoms immobilise prey by disrupting the coagulation cascade in various ways, including via activation or inhibition of coagulation factors [13,14,15,16]. Procoagulant venoms induce clotting by either possessing activated forms of clotting factors in the venom itself or by activating endogenous zymogens, such as prothrombin, Factor V or Factor X [13,15]. Procoagulant venoms ultimately produce large amounts of endogenous thrombin, leading to a well-ordered, fibrin clot. Snake venom metalloproteases (SVMPs) have been shown to be responsible for the prothrombin and Factor X activation produced by *Bothrops* venoms [7,11,17,18], with differential activity between SVMP isoforms in relative prothrombin or Factor X activation [19]. The possession of such toxins makes *Bothrops* venoms deadly, underscoring the need for antivenom treatment for envenomed patients. Depending on species and geographic region, a multitude of different antivenoms is used to treat *Bothrops* envenomations. These antivenoms include, among others, Antivipmyn (Instituto Bioclon, Mexico) [20], Central American polyspecific antivenom (PoliVal-ICP; Instituto Clodomiro Picado, Costa Rica) [21,22], Antivipmyn-Tri (Instituto Bioclon, Mexico) [21,23,24], Soro Antibotrópico (SAB; Instituto Butantan, Brazil) [25], and Bothrofav (MicroPharm, United Kingdom) [21]. Despite antivenom being available to treat envenomations, a fundamental, life-threatening issue with antivenom efficacy arises due to the natural extreme complexity of venoms that have been shaped by a myriad of selection pressures. In fact, diversity among snake venoms (both inter- and intraspecifically) is ubiquitous, with the main driver of venom variation often attributed to diet differences [26,27]. Diet-related selection pressures on venom can include prey type, prey escape potential, and prey retaliation potential [11,28]. However, neutral evolution processes, such as genetic drift, genetic bottleneck, or founder effect, are additional drivers of venom variation [29,30].

Many studies have shown intraspecific venom variation in *B. asper* and *B. atrox* [11,31,32,33]. In fact, venoms from the *Bothrops* genus (specifically *B. asper* and *B. atrox*) are highly malleable to intra- and interspecific venom variation, due to their evolutionary history and geographic range. During the Miocene epoch, between 23–10 million years ago (Mya), the common ancestor of the *Bothrops* + *Bothrocophias* (sister genera to *Bothrops*) clade colonised the then viperid-devoid South America, with the *Bothrops* genus splitting off subsequent to the invasion of South America, where it rapidly diversified [34]. Approximately 3.34 Mya, *B. atrox* and *B. asper* diverged from a common ancestor into separate species, and soon after, *B. asper* reinvaded Middle America in at least two independent dispersal events [35,36]. Today, *B. atrox* occurs throughout South America, from Brazil to Northern South America, while *B. asper* occurs across Mexico, Central, and South America [37,38]. Such a vast geographic range of both species could result in geographically isolated populations under different selection pressures, thereby differentially shaping venom evolution. This is likely the driver of *B. asper* and *B. atrox* venom variation observed in previous studies [11,31,32,36]. Furthermore, evidence of distinct lineages within *B. atrox* suggests the species is probably a complex of multiple species [7,39,40]. This could also be the case for *B. asper*, as previous work suggests [35,41]. Speciation occurs due to different selection pressures acting on each species and, thus, would undoubtedly affect venom composition.

Despite a multitude of antivenoms being available for treating envenomations by species of the same genus, there has not been a standardised comparison of them against the two widest-ranging medically important species in Latin America (*B. asper and B. atrox*), which thus impedes the evidence-based design of clinical management strategies. It is especially important to test the use of antivenoms made for snakes in one region for their cross-reactivity for snakebites in regions that do not have a locally produced antivenom. Use of non-local antivenom is seen in French Guiana, which, due to their lack of a local antivenom, adopted Mexico’s Antivipmyn-Tri to treat snakebites, including *B. atrox* snakebites, in the region [23]. However, the literature on the neutralising ability of Antivipmyn-Tri for French Guiana snakes is scarce [23,24,42,43], and, therefore, there is a knowledge gap regarding its efficacy in treating critically envenomated patients. Cross-species neutralisation has, however, been noted for other antivenoms, such as SAB from Instituto Butantan, which does not contain *B. atrox* in the immunising mixture but has been proven to be effective in neutralising the coagulotoxic venoms of some *B. atrox* populations [11]. It is therefore relevant to assess whether antivenoms available in Latin America are effective in the neutralisation of venoms of *B. asper and B. atrox* from various geographic locations [44].

The aim of this study, therefore, is to investigate venom *in vitro* clotting activity variation and antivenom efficacy across geographically distinct populations of *B. asper* and *B. atrox* using five antivenoms that are currently used to treat *Bothrops* bites. It is hypothesised that there will be venom variation both within and between species and that this will result in differential antivenom efficacy. This knowledge will be fundamental for improving antivenom design in Latin America.

## 2. Results

The spontaneous clotting control of plasma and the positive kaolin control was 459.90 ± 67.13 s (*n* = 4) and 63.08 ± 0.67 s (*n* = 4), respectively. Antivenoms did not have any effect on clotting as antivenom control values were similar to the spontaneous clotting time: Antivipmyn (*n* = 4) = 485.63 ± 54.41 s, PoliVal-ICP (*n* = 4) = 483.15 ± 27.93 s, Antivipmyn-Tri (*n* = 4) = 486.45 ± 20.97 s, Bothrofav (*n* = 4) = 521.45 ± 9.32 s, and SAB (*n* = 4) = 467.08 ± 30.79 s. A Brown–Forsythe ANOVA test revealed no significant differences between antivenom controls and the spontaneous clotting time (F (5.00, 9.21) = 1.13, *p* = 0.41).

**Figure 1:**
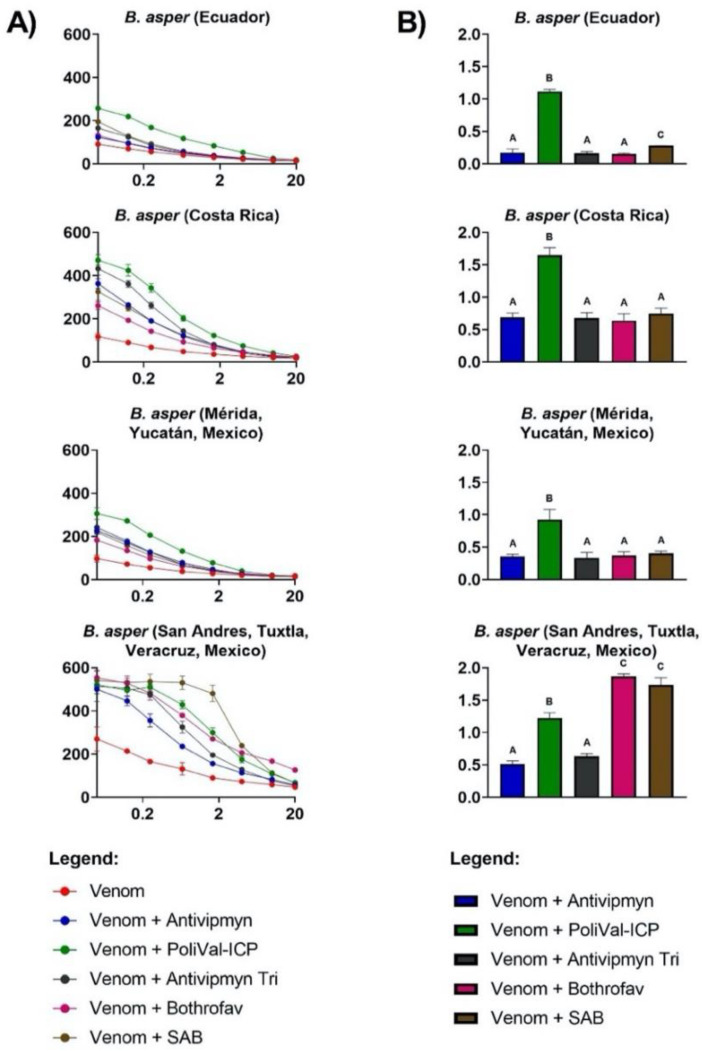
(**A**) Human plasma clotting times (*y*-axis) induced by *Bothrops asper* venom (red line) and venom + antivenom treatments (line colours indicated by the legend) over eight different venom concentrations (μg/mL: 20, 10, 4, 1.66, 0.66, 0.25, 0.125, and 0.05; *x*-axis). The percentage of antivenom in the final cuvette is 0.25%. The graphs’ *x*-axis is displayed in logarithmic view. Each of the eight data points per venom curve is represented by dots (mean value, *n* = 3), with standard deviation error bars. Some error bars are smaller than the data-point symbol. Control values (seconds ± standard deviation) were negative control (spontaneous clotting time of plasma, *n* = 4) = 459.90 ± 67.13, positive control (kaolin, *n* = 4) = 63.08 ± 0.67, and antivenom controls (Antivipmyn (*n* = 4) = 485.63 ± 54.41, PoliVal-ICP (*n* = 4) = 483.15 ± 27.93, Antivipmyn-Tri (*n* = 4) = 486.45 ± 20.97, Bothrofav (*n* = 4) = 521.45 ± 9.32, and SAB (*n* = 4) = 467.08 ± 30.79). See Section 4.3 and Section 4.4 for control methodologies. (**B**) X-fold shift in plasma clotting curves (*y*-axis) of *Bothrops asper* venom incubated with five different antivenoms (bar colours indicated by the legend). X-fold shift values were calculated for each venom by dividing the area under the curve (AUC) for the venom incubated with antivenom clotting time curve by the AUC for the venom clotting time curve, and subtracting the total by 1. Calculated values represent antivenom neutralisation: a value of 0 indicates no neutralisation, while a value of above 0 indicates neutralisation. Values are mean (*n* = 3) ± standard deviation. Letters above bars show statistical significance (*p* < 0.05). Different letters indicate a significant difference between values, while the same letter across multiple bars indicates no significance. The statistical test used was Tukey’s multiple comparisons test, following significant ordinary one-way analysis of variance (ANOVA) results. SAB = Soro Antibotrópico.

**Figure 2:**
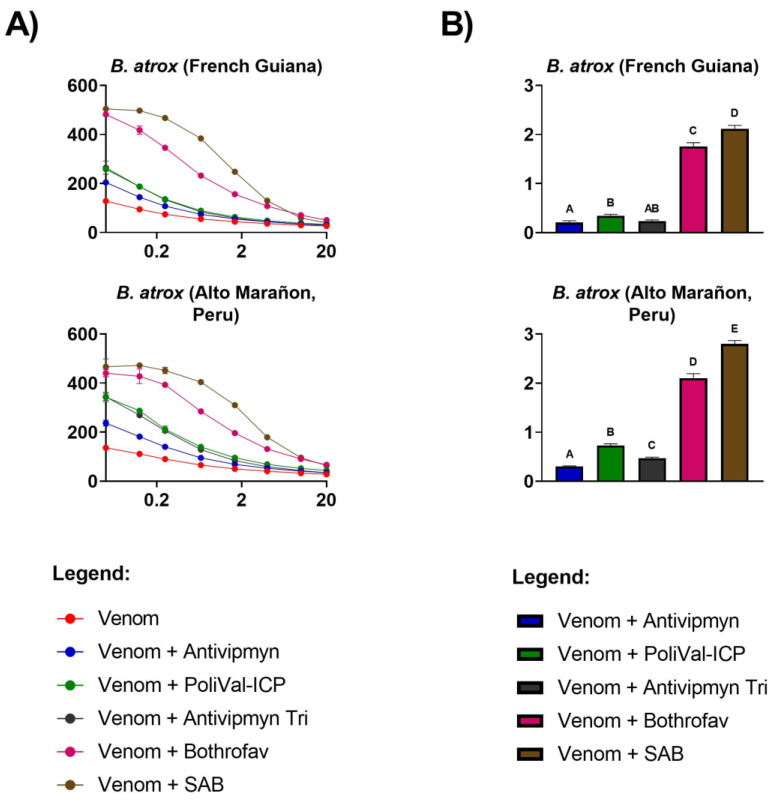
(**A**) Human plasma clotting times (*y*-axis) induced by *Bothrops atrox* venom (red line) and venom + antivenom treatments (line colours indicated by the legend) over eight different venom concentrations (μg/mL: 20, 10, 4, 1.66, 0.66, 0.25, 0.125, and 0.05; *x*-axis). The percentage of antivenom in the final cuvette is 0.25%. The graphs’ *x*-axis is displayed in logarithmic view. Each of the eight data points per venom curve is represented by dots (mean value, *n* = 3), with standard deviation error bars. Some error bars are smaller than the data-point symbol. Control values (seconds ± standard deviation) were negative control (spontaneous clotting time of plasma, *n* = 4) = 459.90 ± 67.13, positive control (kaolin, *n* = 4) = 63.08 ± 0.67, and antivenom controls (Antivipmyn (*n* = 4) = 485.63 ± 54.41, PoliVal-ICP (*n* = 4) = 483.15 ± 27.93, Antivipmyn-Tri (*n* = 4) = 486.45 ± 20.97, Bothrofav (*n* = 4) = 521.45 ± 9.32, and SAB (*n* = 4) = 467.08 ± 30.79). See Section 4.3 and Section 4.4 for control methodologies. (**B**) X-fold shift in plasma clotting curves (*y*-axis) of *Bothrops atrox* venom incubated with five different antivenoms (bar colours indicated by the legend). X-fold shift values were calculated for each venom by dividing the area under the curve (AUC) for the venom incubated with antivenom clotting time curve by the AUC for the venom clotting time curve, and subtracting the total by 1. Calculated values represent antivenom neutralisation: a value of 0 indicates no neutralisation, while a value of above 0 indicates neutralisation. Values are mean (*n* = 3) ± standard deviation. Letters above bars show statistical significance (*p* < 0.05). Different letters indicate a significant difference between values, while the same letter across multiple bars indicates no significance. The statistical test used was Tukey’s multiple comparisons test, following significant ordinary one-way analysis of variance (ANOVA) results. SAB = Soro Antibotrópico.

All venoms exhibited extremely strong procoagulant effects on plasma, with clotting times at 20 µg/mL venom concentration (at which the concentration of all venoms was well into the asymptotic phase and, thus, at maximum clotting velocity), ranging from 13.50 ± 0.85 s for *B. asper* (Ecuador) to 47.20 ± 2.04 s for *B. asper* (San Andres, Tuxtla, (S.A.T.), Mexico) (Table 1, Figure 1A and Figure 2A). Both intra- and interspecific variations were observed. Based upon the area under the curve (AUC) values (Table 1), the relative ranking of the venoms from most potent (smallest AUC) to least potent (largest AUC) was *B. asper* (Yucatán, Mexico) 377.33 ± 9.15; *B. asper* (Ecuador) 384.00 ± 5.86; *B. asper* (Costa Rica) 481.70 ± 18.01; *B. atrox* (French Guiana) 669.70 ± 15.85; *B. atrox* (Alto Marañon (A.M.), Peru) 741.73 ± 5.47; *B. asper* (S.A.T., Mexico) 1,327.33 ± 26.03. Considerable intraspecies variation was observed in *B. asper* AUC values across localities, with *B. asper* (S.A.T., Mexico) AUC being 72% (100 − (377.33/1327.33) * 100) greater (less potent) than *B. asper* (Yucatán, Mexico) (Table 1, Figure 1A). These difference between Mexican *B. asper* AUC values were significant (Tukey’s multiple comparisons test, *p* < 0.0001; Table 2).

Amongst all the antivenoms tested, PoliVal-ICP was the best performing antivenom for all *B. asper* samples except *B. asper* (S.A.T., Mexico; Figure 1). This antivenom significantly induced the highest X-fold shift (slowing of clotting times due to venom neutralisation) among all antivenoms for these samples, with the other antivenoms having very low neutralisation compared to PoliVal-ICP (Table 3). In contrast, *B. asper* (S.A.T., Mexico) was best neutralised by Bothrofav and SAB (X-fold shift = 1.83 ± 0.06 and 1.74 ± 0.11, respectively; Figure 1), and the X-fold shift values were significantly higher than all other antivenoms including PoliVal-ICP (Table 3).

**Figure 3:**
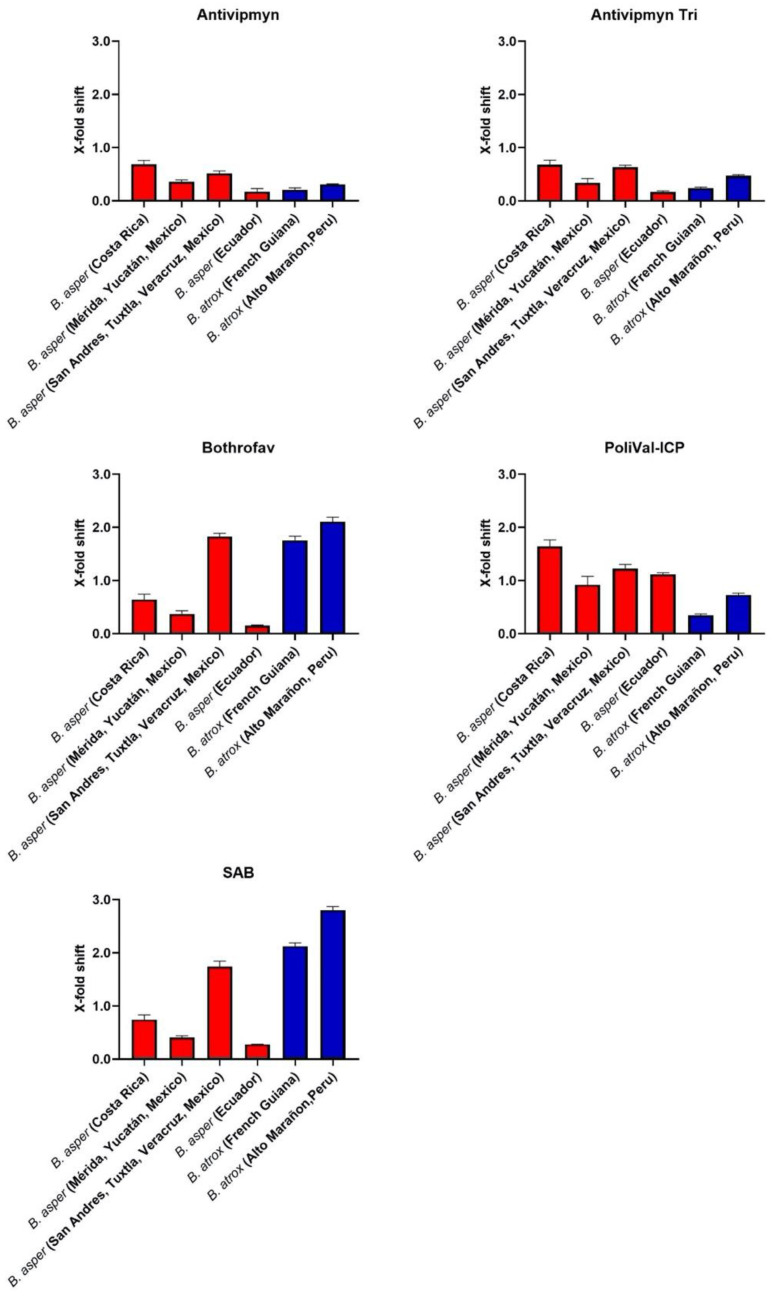
Each bar graph represents the X-fold shift value of each venom when incubated with antivenom (antivenom indicated by the graphs’ title). Note SAB = Soro Antibotrópico. X-fold shift values were calculated for each venom by dividing the area under the curve (AUC) for the venom incubated with antivenom clotting time curve by the AUC for the venom clotting time curve, and subtracting the total by 1. Calculated values represent antivenom neutralisation: a value of 0 indicates no neutralisation, while a value of above 0 indicates neutralisation. Each bar is a mean (*n* = 3) ± standard deviation. Red bars indicate venom samples are from *Bothrops asper*, while blue bars indicate venom samples are from *Bothrops atrox*. Venom sample information is also displayed below each bar. The percentage of antivenom in the final cuvette is 0.25%.

Across all the localities of *B. atrox*, SAB was the best performing antivenom, with the X-fold shift significantly higher than all other antivenoms tested (Figure 2, Table 4). Bothrofav also neutralised French Guiana and A.M. (Peru) localities in a similar way to SAB (Figure 2). All other antivenoms had lower neutralisation compared to the best performing antivenom (Figure 2). Variation in antivenom neutralisation was observed between Mexican *B. asper* samples and between *B. atrox* samples. The S.A.T (Mexico) locality was the best neutralised across all the antivenoms compared to the Yucatán (Mexico) locality (Figure 3.). A similar result was observed for the *B. atrox* samples, with the A.M. (Peru) locality being the best neutralised across all the antivenoms compared to the French Guiana locality (Figure 3).

A clear species-specific pattern of neutralisation was observed for PoliVal-ICP, with all *B. asper* samples better neutralised than *B. atrox* samples (Figure 3). These differences were significant: *B. asper* samples had significantly higher X-fold shift values than *B. atrox* samples, with the exception being *B. asper* (Yucatán, Mexico) as it was not significantly different from *B. atrox* (A.M., Peru; Table 5). A species-specific pattern was also observed for SAB and Bothrofav, which significantly neutralised all *B. atrox* samples to a greater extent than *B. asper* samples, except for *B. asper* (S.A.T., Mexico; Figure 3, Table 5). Antivipmyn displayed a possible geographically specific pattern of neutralisation: all Central American and Mexican samples were better neutralised than the South American samples (Figure 3). These differences were significant: Central American and Mexican samples had significantly higher X-fold shift values than South American samples, with the exception being *B. asper* (Yucatán, Mexico) as it was not significantly different from *B. atrox* (A.M., Peru; Table 6). No clear species-specific or geographically specific pattern of neutralisation was observed for Antivipmyn-Tri (Figure 3).

## 3. Discussion

The aim of this study was to investigate the venom effects of multiple *B. asper* and *B. atrox* localities on human plasma and to ascertain the relative efficacy of five regionally important antivenoms used to treat *Bothrops* bites. It was hypothesised that both intra- and interspecific venom variation would be observed and would result in differential antivenom efficacy. Indeed, the venoms tested differed in coagulotoxic activity both intra- and interspecifically and in antivenom neutralising potential (Figure 1, Figure 2 and Figure 3, Table 1). SAB and Bothrofav performed well against *B. atrox* venoms (Figure 2, Table 4), while PoliVal-ICP neutralised all *B. asper* samples considerably better than other antivenoms, except for *B. asper* (S.A.T., Mexico; Figure 1, Table 3). PoliVal-ICP, Bothrofav, and SAB showed species-specific patterns of neutralisation, while Antivipmyn showed a geographically specific pattern of neutralisation (Figure 3).

Venom from all *Bothrops* tested displayed potent procoagulant activity on our assays (Figure 1A and Figure 2A, Table 1). In prey animals, this procoagulant action results in rapid incapacitation by the induction of stroke. However, due to the dilution of the venom into a much large blood volume, thrombotic strokes in humans from *Bothrops* envenomations are rare but have been reported [45]. The more common outcome of the out-of-control activation of the clotting cascade is unclottable blood and systemic bleeding [7,8,25,46], due to venom induced consumptive coagulopathy [47]. An exception to this general trend is the venom of *B. lanceolatus*, endemic to the Caribbean island of Martinique, which is more likely than other *Bothrops* species to cause severe thrombosis and infarctions [48].

Despite the consistent procoagulant activity of all species, our study found significant differences in AUC values (representing clotting activity; smaller AUC values indicate greater procoagulant activity, and vice versa) across different localities (ANOVA: F (5, 12) = 1644.00, *p* < 0.0001; Figure 1A and Figure 2A, Table 1 and Table 2). This result is consistent with previous literature, which found *B. asper* and *B. atrox* venom activity to vary between different localities [11,31,32,33].

Clotting times induced by *B. asper* venoms were similar across Costa Rica, Ecuador, and Yucatán (Mexico) localities. The S.A.T. (Mexico) locality, however, had considerably slower clotting times (Figure 1A, Table 1). Unlike the other samples, our *B. asper* Mexican samples were individual venoms rather than pooled venoms (Table 7). Individual venom variation has been observed in multiple snake species [49,50,51]; thus pooled venoms are favoured as they help account for any possible variation between individuals. Therefore, in the present study, individual variation cannot be ruled out as contributing to the observed variation in venom potency and antivenom efficacy. Future work should investigate whether the differences between Mexican *B. asper* samples in this study are due to differences in venoms between individual snakes or differences in venom between populations of snakes from different localities. If the latter, drivers of population variation should be investigated through natural history observations.

As with procoagulant activity, variation amongst antivenom efficacy was evident, both between and within species, and across different antivenoms (Figure 1, Figure 2 and Figure 3). A possible geographical pattern of antivenom neutralisation across both *Bothrops* species tested was observed for Antivipmyn, whereby most Central American and Mexican venom samples, with the exception being *B. asper* (Yucatán, Mexico), were significantly more neutralised than South American venom samples (Figure 3, Table 6). This geographical pattern is evident, as the only South American *B. asper* sample (*B. asper* (Ecuador)) was the least neutralised *B. asper* sample by this antivenom. This result indicates different toxin compositions between South American and Central/North American *B. asper*, possibly driven by founder effects following the invasion of Central America from South America and the resulting genetic bottleneck imposed by the transit through the very narrow Isthmus of Panama. Furthermore, the venom variation observed may correspond with the Central American and Mexican *B. asper* samples in this study being of a different lineage than the Ecuador *B. asper* sample. Two divergent lineages of *B. asper* have been recognised: one comprising snakes from southwestern Ecuador and the Caribbean coast of Middle America (Clade A), and the other comprising snakes from northwestern South America and Pacific regions of Costa Rica and Panama (Clade B) [35]. In fact, venom variation has been observed in Costa Rican *B. asper* from Pacific and Caribbean regions [32,33]. Thus, differences observed between *B. asper* samples may reflect their geographic location and evoluntionary history.

A clear species-specific pattern was observed for PoliVal-ICP, since most *B. asper* venom samples were significantly more neutralised than *B. atrox* venom samples (Figure 3, Table 5). This suggests a high cross-reactivity of the antivenom to *B. asper* localities besides just the *B. asper* locality of Costa Rica, which is used in the antivenom immunizing mixture of PoliVal-ICP (see Table 8). PoliVal-ICP also neutralised Mexican *B. asper* samples better than Antivipmyn (Tukey’s multiple comparisons test, *p* < 0.0001; Figure 1, Table 3), which is another antivenom with *B. asper* venom in the immunising mixture (Table 8). However, it is important to note that only two individual venom samples from Mexico were used in the study; thus, more comprehensive sampling must be undertaken before evidence-based medical recommendations can be made. Furthermore, it is important to note that Antivipmyn significantly neutralised *B. asper* from S.A.T. (Mexico) better than *B. asper* from Yucatán (Mexico) (Tukey’s multiple comparisons test: *p* = 0.0158; Figure 3). Thus, not only do these venoms differ in their coagulotoxic profiles, but these differences also reflect antivenom neutralisation. To further elucidate the efficacy of Antivipmyn and the drivers of this variation, as seen amongst Mexican samples, more work needs to be done in order to better inform on treatment strategies for the envenomed patient.

SAB and Bothrofav also showed species-specific effects by neutralising all *B. atrox* samples to a greater extent than *B. asper* samples, with the exception being the high neutralisation of Bothrofav to the S.A.T. (Mexico) locality (Figure 3, Table 5). Despite SAB antivenom not having *B. atrox* in the immunising mixture (but using related species; see Table 8), its strong cross-reactivity with *B. atrox* is consistent with other studies [11]. The cross-reactivity of antivenoms similar to SAB (produced with similar species) with *B. atrox* has also been observed [52]. In this regard, proteomic analysis of several *Bothrops* venoms has been reported, such as the protein composition of the Peruvian *B. atrox* included in this work, and correlated with their main pharmacological properties [53]. It has become clear that a low number of toxic compounds belonging to the predominant protein families (snake venom metalloproteases, serine proteases, phospholipases A2 and l-amino acid oxidases), among others, compose the venom of *Bothrops* species [36,53]. Thus, data of the literature and results obtained in this study provide evidence of the degree of antigenic conservation among *Bothrops* snakes.

On the other hand, this is the first time the strong cross-reactivity of Bothrofav antivenom against *B. atrox* venoms has been shown. The high neutralisation of Bothrofav to *B. asper* (S.A.T., Mexico), but not *B. asper* (Yucatán, Mexico) (Figure 1 and Figure 3), further indicates important differences in venom composition between these samples. A previous study has described the lack of efficacy of a first-generation Bothrofav antivenom for neutralising *in vitro* coagulant activity of venoms of *B. asper* and *B. atrox* [54]. It is likely that changes have been introduced in the manufacturing of more recent batches of this antivenom, which would explain its efficacy in the neutralisation of procoagulant activity of *B. atrox* venom, as shown in our work.

Overall, these results help inform antivenom treatment plans for regions without antivenoms containing local snake venom in the immunising mixture. Antivipmyn-Tri is currently used in French Guiana to treat snakebites [23], although our results show it has low neutralising efficacy for the coagulotoxic effects of *B. atrox* inhabiting French Guiana and the other *B. atrox* venoms tested in this study. These results are congruent with a recent retrospective study that looked at the efficacy of Antivipmyn-Tri and concluded that compared to patients receiving no antivenom, it did not benefit patient recovery from coagulopathy as a result of envenomation [24]. Bothrofav and SAB perform better at neutralising the procoagulant activity of *B. atrox* from French Guiana. Furthermore, Bothrofav antivenom is used to treat *B. lanceolatus*, a species inhabiting the Caribbean island of Martinique. However, in comparison to other antivenoms used, it showed high efficacy in neutralising the coagulotoxic effect of all *B. atrox* studied, as well as *B. asper* from S.A.T. (Mexico) (Figure 1, Figure 2 and Figure 3). This finding is interesting in the light of the low procoagulant activity described for the venom of *B. lanceolatus* [54,55]. However, it must be noted that these studies examined the coagulation of citrated plasma without recalcifying it. It has been shown that recalcification is essential to ascertain procoagulant toxins [11]. Thus, the previous lack of demonstrated procoagulant activity of *B. lanceolatus* in laboratory studies is likely due to methodological conditions, especially in light of the devastating procoagulant activity that this species has been documented as having in clinical reports [45,48]. Furthermore, the nature of venom components in *B. lanceolatus* that elicit the antibody response capable of neutralising the procoagulant effect of *B. atrox* venom deserves further investigation.

It is important to note the limitations of the study so that appropriate conclusions can be drawn and future work mapped out. Firstly, all antivenom efficacy results are based on the ability of the antivenom to neutralise the coagulotoxic effects of the venom on platelet-poor plasma and not other venom effects, such as the activation and inhibitory effects on platelet aggregation [17,56,57,58], and myotoxicity [59,60]. Thus, even if an antivenom neutralises procoagulant coagulotoxic effects, other medically important venom effects may not be neutralised. Secondly, another caveat in the study is the use of some antivenoms beyond their expiry date. However, it has been documented that antivenoms, especially freeze-dried ones, maintain their neutralising effect well beyond their expiry date, with studies showing that their efficacy was retained even decades later (up to 60 years in one study) [61,62]. Furthermore, one study showed no significant change in specific antibody concentration when liquid-formulated antivenoms were stored at 4 °C vs. ambient temperature [63], hence showing how robust some antivenoms can be. Thus, while a quantitative reduction in their neutralising ability might occur, as observed in O’Leary, Kornhauser, Hodgson and Isbister [62], this does not affect the main qualitative conclusions reached in this study regarding the species-specific and geographically specific patterns of neutralisation by the antivenoms. Thirdly, proteomic work was not performed in this study; thus, differences in venom function and resultant antivenom efficacy cannot be discussed in the context of differences in proteins. Proteomic work should be investigated in futures studies to elucidate the differences in venom components and how these impact antivenom efficacy.

Another important caveat of the present study is that the experiments are *in vitro*. *In vivo* systems are far more complicated (e.g., involving multiple physiological systems and feedback loops) and further studies should confirm the efficacy of the antivenoms used in this study on *in vivo* models, assessing, for example, the neutralisation of hemorrhagic and defibrinogenating activities. However, while success in this study needs to be corroborated with *in vivo* follow-up work, it is unlikely for an antivenom to perform better *in vivo* than *in vitro* where variables are removed and the antivenom is given an ideal situation (i.e., in close proximity with no flow) to bind to the toxins. Thus, antivenoms that failed to neutralise a venom under our study conditions would be exceedingly unlikely to neutralise the venom in a more dynamic *in vivo* scenario. Therefore, indications of poor performance obtained under the ideal conditions are strongly suggestive of poor performance in real-world scenarios. Conversely, for antivenoms that worked well against a particular venom in the *in vitro* assays of this study, it is critical that such results are confirmed with *in vivo* assays and clinical trials before recommendations are made regarding therapeutic use. Therefore, while the overall patterns of antivenom efficacy observed in this current study are important and informative, additional work *in vivo* and in clinical trials are required before reaching more conclusive therapeutic recommendations. 

In conclusion, the results of the present study are important not only to evolutionary biologists interested in venom variation, but also to antivenom manufacturers and clinicians treating snakebites in Latin America. The intraspecific variation of *Bothrops* and other snake species needs to be considered when developing and using antivenoms. Studies like the present add to the body of knowledge required for the evidence-based design of clinical management strategies.

## 4. Materials and Methods

### 4.1. Snake Venom Sample Preparation

All work was performed under University of Queensland IBSC approval #IBC134BSBS2015. All snake venom samples were sourced from either venom suppliers or the Toxin Evolution Lab collection at the University of Queensland. Samples were stored in lyophilised form until use. In this study, six venom samples from the *Bothrops* genus (four *B. asper* and two *B. atrox* samples), including multiple geographic localities, were used (Table 7). It was not a concern that some venom samples were from captive snakes and some from wild snakes, as studies on *Bothrops* have shown captivity does not influence venom variation [64]. For experiments, lyophilised venom samples were solubilised with deionised H_2_O and their protein concentration measured in triplicate using Thermo Fisher Scientific™ NanoDrop 2000 (Waltham, MA, USA) at 280 nm wavelength. Venom samples were then made into working stocks of 1 mg/mL 1:1 deionised H_2_O:glycerol and stored (frozen) at −80 °C until required, whereby they were then stored (unfrozen) at −20 °C. To avoid protein degradation, glycerol was used to stabilise the enzymes, thus preventing degradation due to autocatalysis.

### 4.2. Human Plasma

All human plasma work was performed under University of Queensland Biosafety Approval #IBC134BSBS2015 and Human Ethics Approval #2016000256. Surplus human platelet-poor plasma (3.2% citrated) was supplied by the Australian Red Cross (44 Musk Street, Kelvin Grove, Queensland 4059) under research approval #16-04QLD-10 and stored at −80 °C until required. Platelet-poor plasma, as opposed to platelet-rich plasma, was used due to its long-term stability. Furthermore, platelet-poor plasma is commonly used in coagulation studies, as platelets can interact with reagents and shorten clotting times [65]. For the present study, one batch of platelet-poor plasma (Batch # A540019384586, O positive, collected 3 May 2019) was defrosted at 37 °C and aliquoted into 1.5 mL tubes in a laminar flow hood to avoid contamination. Aliquots were flash-frozen with liquid nitrogen and stored at −80 °C. Platelet-poor plasma was defrosted for 5 min in a 37 °C water bath before being used in assays (see Section 4.3 and Section 4.4).

### 4.3. Venom-Induced Coagulation

Coagulation assays were performed on a Stago STA-R Max haemostasis analyser (Stago, Asnières sur Seine, France) using a previously established assay [66,67,68]. The clotting time of platelet-poor plasma was measured in triplicate at eight different venom concentrations (μg/mL: 0.05, 0.125, 0.25, 0.66, 1.66, 4, 10, and 20), using all venoms (Table 7). For testing, venom stock was diluted 1:10 using Owren Koller (OK) buffer (Stago Cat #00360) to make a 0.1 mg/mL solution. This working solution was added into the analyser. For the 1:1 dilution (20 μg/mL), the machine added 50 μL of the venom sample into a cuvette with cofactors (50 μL of 25 mM CaCl_2_ (Stago Cat# 00367), 50 μL of phospholipid (Stago Cat# 00597), and 25 µL of OK buffer). The cuvette was then incubated for 120 s at 37 °C. After incubation, 75 µL of platelet-poor plasma was added into the incubated venom mix (final cuvette volume = 250 µL) and clotting time was automatically measured via a mechanical, viscosity-based system. For subsequent venom concentrations, the volume of the venom sample added into the cuvette was adjusted and additional OK buffer was added to maintain a final cuvette volume of 250 µL. Positive and negative controls were performed to ensure the health of the plasma and reagents. A kaolin test (similar to aPTT (activated partial thromboplastin time)) was used as the positive control: 50 µL of platelet-poor plasma incubated with 50 µL kaolin (Stago kit; Cat# 00597), 50 µL phospholipid, and 50 µL OK buffer for 120 s. Then, 25 mM CaCl_2_ was added postincubation to initiate clotting. The negative control used was the spontaneous clotting time of plasma, in which the same volume of reagents was used as the venom coagulation assay, except a 1:10 diluted (diluent = OK buffer) 50% deionised H_2_O/50% glycerol sample was used instead of the venom sample. Dilution series were not performed for controls. All tests were performed in triplicate, and clotting time was measured up until 999 s (machine maximum reading time).

### 4.4. Antivenom Neutralising Efficacy

Five antivenoms that are used to treat *Bothrops* envenomings were included in the study (Table 8). Expired antivenoms were not a concern, as antivenoms have been shown to still be effective after their expiry date [61,62]. In particular, freeze-dried antivenoms are highly stable for many years after the official expiration date. Antivipmyn and Antivipmyn-Tri were both freeze-dried antivenoms, while all other antivenoms used were in liquid form. All antivenoms were centrifuged at 12,000 relative centrifugal force (RCF) on an Allegra™ X-22R Centrifuge (Lot#982501, Beckman Coulter, Brea, CA, USA) at 4 °C for 10 min; then, the supernatant was removed and filtered with a 0.45 µm filter, aliquoted (1 mL into 1.5 mL tubes), and stored at 4 °C. Antivenom coagulation assays were performed for each antivenom to determine their potential to neutralise the coagulotoxic effects of each venom. These assays were performed on a Stago STA-R Max haemostasis analyser, as previously described [66,67,68], whereby the same procedure as the venom coagulation assays (see Section 4.3) was used, except the 25 μL OK buffer was replaced with 25 μL of 2.5% (*v/v*) antivenom (final cuvette concentration of 0.25%). Prior to testing, the antivenom was prepared to 2.5% by diluting with OK buffer. A negative control for antivenom assays was performed, in which a 1:10 diluted (diluent = OK buffer) 50% deionised H_2_O/50% glycerol sample was used instead of the venom sample. This control was performed to see if the antivenom had any effect on clotting.

### 4.5. Statistical Analyses

All raw data is available in Supplementary Dataset S1. All statistical analyses were performed in GraphPad PRISM 8.1.1 (GraphPad Prism Inc., La Jolla, CA, USA). A *p*-value of less than 0.05 was considered significant. When reporting *p*-values and the associated information, the following abbreviations are used: *p* means *p*-value and F means F-value. When reporting F-values, the degrees of freedom are shown in brackets. All results in the study are shown as the mean ± standard deviation.

Eight-point dilution curves, showing the clotting time of each venom and venom + antivenom across a venom concentration gradient, were graphed using GraphPad PRISM 8.1.1. To more clearly view the data, the *x*-axis for eight-point dilution curves was presented in logarithmic view. To calculate the neutralising capacity of each venom, an area under the curve (AUC) value was obtained in GraphPad PRISM 8.1.1 for each of the venom curves and venom + antivenom curves. An X-fold shift value was calculated with the following formula: $\frac{\mathrm{venom}\;+\;\mathrm{antivenom}\;\mathrm{AUC}\;}{\mathrm{Venom}\;\mathrm{AUC}}-1$; a value of 0 indicates no neutralisation and a value above 0 indicates neutralisation. Bar graphs of X-fold shift values were graphed using GraphPad PRISM 8.1.1.

Venom sample AUC values were compared using an ordinary one-way analysis of variance (ANOVA; Table 2). Within each venom, X-fold shifts for different antivenoms were also statistically compared using an ordinary one-way ANOVA (Table 3 and Table 4). Assumptions of the normality of residuals and the homodescasity of residuals were checked using quantile–quantile plots, Shapiro–Wilk and Brown–Forsythe tests. The posthoc test used was Tukey’s multiple comparisons test, in which multiplicity adjusted *p*-values were used that accounted for multiple comparisons. The same statistical procedure was followed to statistically compare X-fold shifts between different venoms within each antivenom (Table 5 and Table 6). To test if antivenoms had any effect on clotting times, the clotting times of antivenom controls were statistically compared to the spontaneous control using the Brown–Forsythe ANOVA. A Brown–Forsythe ANOVA was used as the data violated the assumption of the homodescasity of residuals. A Welch ANOVA was also performed alongside the Brown–Forsythe test due to uncertainty as to which test is best. Despite the *p*-value being barely significant (*p* = 0.0485), all posthoc tests (Dunnett’s T3 multiple comparisons test) were extremely not-significant (ranging from *p* = 0.4038 to *p* = 0.9997); thus, it was deemed that there was no statistical difference between antivenom control and spontaneous control clotting times.

## Figures and Tables

**Table 1:** Venom-induced (20 μg/mL) clotting time (seconds) and area under the curve (AUC) values for *Bothrops* venom samples. All values are mean (*n* = 3) ± standard deviation.

Venom	20 μg/mL Clotting Time	AUC Value
*B. asper* (Costa Rica)	17.73 ± 2.17	481.70 ± 18.01
*B. asper* (Ecuador)	13.50 ± 0.85	384.00 ± 5.86
*B. asper* (Mérida, Yucatán, Mexico)	14.27 ± 1.12	377.33 ± 9.15
*B. asper* (San Andres, Tuxtla, Veracruz, Mexico)	47.20 ± 2.04	1327.33 ± 26.03
*B. atrox* (French Guiana)	26.80 ± 0.96	669.70 ± 15.85
*B. atrox* (Alto Marañon, Peru)	28.30 ± 0.66	741.73 ± 5.47

**Table 2:** *p*-values of Tukey’s multiple comparisons tests for an ordinary one-way analysis of variance (ANOVA; F (5, 12) = 1644.00, *p* < 0.0001) comparing area under the curve (AUC) values of venom samples. A significant result is *p* < 0.05. X indicates no valid comparison.

	*B. asper* (Costa Rica)	*B. asper* (Ecuador)	*B. asper* (Mérida, Yucatán, Mexico)	*B. asper* (San Andres, Tuxtla, Veracruz, Mexico)	*B. atrox* (French Guiana)	*B. atrox* (Alto Marañon, Peru)
*B. asper* (Costa Rica)	X	<0.0001	<0.0001	<0.0001	<0.0001	<0.0001
*B. asper* (Ecuador)	X	X	0.9936	<0.0001	<0.0001	<0.0001
*B. asper* (Mérida, Yucatán, Mexico)	X	X	X	<0.0001	<0.0001	<0.0001
*B. asper* (San Andres, Tuxtla, Veracruz, Mexico)	X	X	X	X	<0.0001	<0.0001
*B. atrox* (French Guiana)	X	X	X	X	X	0.0010
*B. atrox* (Alto Marañon, Peru)	X	X	X	X	X	X

**Table 3:** *p*-values of Tukey’s multiple comparisons tests comparing the X-fold shift values of different antivenoms for all *Bothrops asper* samples. Tukey’s multiple comparisons tests were performed on each *B. asper* locality X-fold shift dataset following significant ordinary one-way analysis of variance (ANOVA) results: *B. asper* (Costa Rica; F (4, 10) = 61.52, *p* < 0.0001), *B. asper* (Ecuador; F (4, 10) = 460.40, *p* < 0.0001), *B. asper* (Mérida, Yucatán, Mexico; F (4, 10) = 25.05, *p* < 0.0001) and *B. asper* (San Andres, Tuxtla, Veracruz, Mexico; F (4, 10) = 209.90, *p* < 0.0001). A significant result is *p* < 0.05. SAB = Soro Antibotrópico.

	*B. asper* (Costa Rica)	*B. asper* (Ecuador)	*B. asper* (Mérida, Yucatán, Mexico)	*B. asper* (San Andres, Tuxtla, Veracruz, Mexico)
Antivipmyn vs. PoliVal-ICP	<0.0001	<0.0001	<0.0001	<0.0001
Antivipmyn vs. Antivipmyn-Tri	>0.9999	0.9996	0.9984	0.3096
Antivipmyn vs. Bothrofav	0.9710	0.9667	0.9993	<0.0001
Antivipmyn vs. SAB	0.9437	0.0169	0.9525	<0.0001
PoliVal-ICP vs. Antivipmyn-Tri	<0.0001	<0.0001	<0.0001	<0.0001
PoliVal-ICP vs. Bothrofav	<0.0001	<0.0001	0.0001	<0.0001
PoliVal-ICP vs. SAB	<0.0001	<0.0001	0.0002	<0.0001
Antivipmyn-Tri vs. Bothrofav	0.9848	0.9916	0.9851	<0.0001
Antivipmyn-Tri vs. SAB	0.9146	0.0125	0.8598	<0.0001
Bothrofav vs. SAB	0.6759	0.0066	0.9882	0.5859

**Table 4:** *p*-values of Tukey’s multiple comparisons tests comparing the X-fold shift values of different antivenoms for both *Bothrops atrox* samples. Tukey’s multiple comparisons tests were performed on each *B. atrox* locality X-fold shift dataset, following significant ordinary one-way analysis of variance (ANOVA) results: *B. atrox* (French Guiana; F (4, 10) = 993.80, *p* < 0.0001), and *B. atrox* (Alto Marañon, Peru; F (4, 10) = 1226.00, *p* < 0.0001).

	*B. atrox* (French Guiana)	*B. atrox* (Alto Marañon, Peru)
Antivipmyn vs. PoliVal-ICP	0.0408	<0.0001
Antivipmyn vs. Antivipmyn-Tri	0.9318	0.0266
Antivipmyn vs. Bothrofav	<0.0001	<0.0001
Antivipmyn vs. SAB	<0.0001	<0.0001
PoliVal-ICP vs. Antivipmyn-Tri	0.1344	0.0013
PoliVal-ICP vs. Bothrofav	<0.0001	<0.0001
PoliVal-ICP vs. SAB	<0.0001	<0.0001
Antivipmyn-Tri vs. Bothrofav	<0.0001	<0.0001
Antivipmyn-Tri vs. SAB	<0.0001	<0.0001
Bothrofav vs. SAB	<0.0001	<0.0001

**Table 5:** *p*-values of Tukey’s multiple comparisons tests comparing X-fold shift values between venoms within an antivenom. Tukey’s multiple comparisons tests were performed on the PoliVal-CP, SAB (Soro Antibotrópico) and BothroFav X-fold shift datasets, following significant ordinary one-way analysis of variance (ANOVA) results: PoliVal-ICP (F (5, 12) = 71.58, *p* < 0.0001), SAB (F (5, 12) = 643.40, *p* < 0.0001), and Bothrofav (F (5, 12) = 389.90, *p* < 0.0001). A significant result is *p* < 0.05. Note that not all Tukey’s multiple comparisons are shown in this table.

Species	Antivenom	*B. asper* (Costa Rica)	*B. asper* (Mérida, Yucatán, Mexico)	*B. asper* (San Andres, Tuxtla, Veracruz, Mexico)	*B. asper* (Ecuador)
*B. atrox* (French Guiana)	PoliVal-ICP	<0.0001	<0.0001	<0.0001	<0.0001
	SAB	<0.0001	<0.0001	0.0002	<0.0001
	Bothrofav	<0.0001	<0.0001	0.8651	<0.0001
*B. atrox* (Alto Marañon, Peru)	PoliVal-ICP	<0.0001	0.1637	0.0003	0.0023
	SAB	<0.0001	<0.0001	<0.0001	<0.0001
	Bothrofav	<0.0001	<0.0001	0.0065	<0.0001

**Table 6:** *p*-values of Tukey’s multiple comparisons tests comparing X-fold shift values between Central American/Mexican and South American venoms within the antivenom Antivipmyn. Tukey’s multiple comparisons tests were performed on the Antivipmyn X-fold shift dataset, following significant ordinary one-way analysis of variance (ANOVA) results: Antivipmyn (F (5, 12) = 50.92, *p* < 0.0001). A significant result is *p* < 0.05. Note that not all Tukey’s multiple comparisons are shown in this table.

		Central America and Mexico		
		*B. asper* (Costa Rica)	*B. asper* (Mérida, Yucatán, Mexico)	*B. asper* (San Andres, Tuxtla, Veracruz, Mexico)
South America	*B. asper* (Ecuador)	<0.0001	0.0044	<0.0001
	*B. atrox* (French Guiana)	<0.0001	0.0213	<0.0001
	*B. atrox* (Alto Marañon, Peru)	<0.0001	0.7904	0.0019

**Table 7:** List of species, localities, abbreviations, and additional information if known (age, sex, habitat, and whether venoms were from wild-caught or captive-born snakes, single individuals or multiple individuals (pooled)). Captive-born samples originate from snakes from the specified locality. N.A. = nonapplicable.

Species	Locality	Abbreviation	Additional Information
*Bothrops asper*	Costa Rica	N.A.	Pooled from adults collected in the Pacific region (*n* = 40)
*Bothrops asper*	Ecuador	N.A.	Pooled from captive-born adult males (*n* = 2)
*Bothrops asper*	Mérida, Yucatán, Mexico	Yucatán, Mexico	Adult (individual)
*Bothrops asper*	San Andres, Tuxtla, Veracruz, Mexico	S.A.T, Mexico	Adult (individual)
*Bothrops atrox*	French Guiana	N.A.	Pooled from captive-born and wild adults (male + females, *n* = 66)
*Bothrops atrox*	Alto Marañon, Peru	A.M., Peru	Pooled from wild adults caught in the Amazon rainforest and kept in captivity (*n* values not supplied)

**Table 8:** Antivenoms used and their relevant information. Information was obtained from package inserts, scientific literature (cited in the table), and/or correspondence with researchers. Species names may have changed since production of the antivenom.

Antivenom	Lot # and Expiry	Immunising Mixture *	Preparation	Neutralising Potency (Specified by Manufacturer)
Antivipmyn^®^, Instituto Bioclon, Mexico	Lot: B-6F-16; Exp: October 2010	*Bothrops asper* (Mexico), *Crotalus simus simus* (Mexico)	Polyspecific equine F(ab’)_2_ preparation	1 vial neutralises >780 LD50 of *Bothrops* sp. and >790 of *Crotalus* sp.
Antivipmyn-Tri^®^, Instituto Bioclon, Mexico	Lot: B-4F-13; Exp: 28 June 2009	*Bothrops asper* (Colombia), *Crotalus simus simus* (Mexico), and *Lachesis muta* (Not stated)	Polyspecific equine F(ab’)_2_ preparation	Unknown
Soro Antibotrópico (SAB)^®^, Instituto Butantan, Brazil	Lot: 1305077; Exp: May 2016	*Bothrops jararaca* (Brazil) (50%), *Bothrops jararacussu* (Brazil) (12.5%), *Bothrops neuwiedi* (Brazil) (12.5%), *Bothrops alternatus* (Brazil) (12.5%), *Bothrops moojeni* (Brazil) (12.5%)	Polyspecific equine IgG F(ab′)_2_ preparation	1 mL neutralises 5 mg venom of *Botrops jararaca* (Brazil)
Central American polyspecific antivenom (PoliVal-ICP)^®^, Instituto Clodomiro Picado, Universidad de Costa Rica	Lot: 5720416; Exp: April 2021	*Bothrops asper* (Costa Rica), *Crotalus simus* (Costa Rica), and *Lachesis stenophrys* (Costa Rica) [69]	Polyspecific equine whole IgG—purified by caprylic acid fractionation [69]	1ml neutralises 3 mg venom of *Bothrops asper* (Costa Rica)
Bothrofav^®^, MicroPharm, United Kingdom	Lot P4AP61V; Exp: October 2020	*Bothrops lanceolatus*	Monospecific equine F(ab′)_2_ preparation	Unknown

* The species of snakes whose venom was used for antivenom production.

## Data Availability

The data presented in this study are available in Supplementary Dataset S1.

## Supplementary Materials

The following are available online at https://www.mdpi.com/2072-6651/13/2/78/s1, Dataset S1: Raw data.
